# Association of Neural Connectome With Early Experiences of Abuse in Adults

**DOI:** 10.1001/jamanetworkopen.2022.53082

**Published:** 2023-01-26

**Authors:** Mayuresh S. Korgaonkar, Isabella A. Breukelaar, Kim Felmingham, Leanne M. Williams, Richard A. Bryant

**Affiliations:** 1 Brain Dynamics Centre, The Westmead Institute for Medical Research, University of Sydney, Westmead, New South Wales, Australia; 2 Discipline of Psychiatry, Sydney Medical School, Westmead, New South Wales, Australia; 3 School of Psychology, University of New South Wales, Sydney, Australia; 4 Discipline of Psychological Science, University of Melbourne, Melbourne, Australia; 5 Department of Psychiatry and Behavioral Sciences, Stanford University, Stanford, California; 6 Sierra-Pacific Mental Illness Research, Education, and Clinical Center, Veterans Affairs Palo Alto Health Care System, Palo Alto, California

## Abstract

**Importance:**

More than 10% of children experience sexual, physical, or emotional abuse, and abuse experienced during sensitive neurodevelopmental periods is associated with a greater risk of psychiatric disorders.

**Objective:**

To investigate the extent to which a history of abuse is associated with alterations in the intrinsic functional connectome of the adult brain independent from the restriction of associated psychiatric conditions.

**Design, Setting, and Participants:**

This cohort study assessed data from 768 adult participants from the greater Sydney, Australia, area who were included in the study without diagnostic restrictions and categorized based on a history of childhood sexual, physical, and/or emotional abuse. Data were collected from January 1, 2009, to December 31, 2015; data analysis was performed from October 1, 2020, to March 31, 2022.

**Main Outcomes and Measures:**

Outcomes were structured psychiatric interview responses, self-report of the frequency and extent of various types of negative experiences in childhood and adolescence, and intrinsic functional connectivity derived from 5 functional magnetic resonance imaging tasks and estimated among 436 brain regions, comprising intranetwork and internetwork connectivity of 8 large-scale brain networks.

**Results:**

Among the 647 individuals with usable data (330 female [51.0%]; mean [SD] age, 33.3 [12.0] years; age range, 18.2-69.2 years), history of abuse was associated with greater likelihood of a current psychiatric illness (odds ratio, 4.55; 95% CI, 3.07-6.72; *P* < .001) and with greater depressive, anxiety, and stress symptoms (mean difference, 20.4; 95% CI, 16.1-24.7; *P* < .001). An altered connectome signature of higher connectivity within somatomotor, dorsal, and ventral attention networks and between these networks and executive control and default mode networks was observed in individuals with a history of abuse experienced during childhood (n = 127) vs those without a history of abuse (n = 442; mean difference, 0.07; 95% CI, 0.05-0.08; familywise, Bonferroni-corrected *P* = .01; Cohen *d* = 0.82) and compared with those who experienced abuse in adolescence (n = 78; mean difference, 0.06; 95% CI, 0.04-0.08]; familywise, Bonferroni-corrected *P* < .001; Cohen *d* = 0.68). Connectome alterations were not observed for those who experienced abuse in adolescence. Connectivity of this signature was transdiagnostic and independent of the nature and frequency of abuse, sex, or current symptomatic state.

**Conclusions and Relevance:**

Findings highlight the associations of exposure to abuse before and during adolescence with the whole-brain functional connectome. The experience of child abuse was found to be associated with physiologic changes in intrinsic connectivity, independent of psychopathology, in a way that may affect functioning of systems responsible for perceptual processing and attention.

## Key Points

**Question:**

Is history of abuse associated with the intrinsic functional connectome of the adult brain independent of current psychiatric illness?

**Findings:**

In this cohort study of 768 participants, individuals with abuse experienced during childhood (but not adolescence) demonstrated an altered connectome of greater functional connectivity associated with somatomotor and dorsal-ventral attention brain networks, irrespective of current diagnosis or symptom state.

**Meaning:**

These findings suggest that a history of child abuse is associated with altered functioning of systems responsible for perceptual processing and attention, and these findings were transdiagnostic.

## Teaser

This cohort study investigates the associations between experiences of abuse in childhood and alterations in whole-brain intrinsic functional connectivity in adulthood without the restriction of psychiatric disorder.

## Introduction

The experience of sexual, physical, or emotional abuse as a child occurs in 5% to 15% of children and substantially increases the risk and severity of psychiatric disorder.^1,2,3^ Childhood abuse is also associated with earlier onset of psychiatric illness, increased comorbidity, higher risk of suicide, and poorer treatment response than seen in nonabused individuals with the same diagnoses.^4^ It is hypothesized that abuse during childhood triggers a cascade of physiologic processes that impact brain structure and function,^5^ which is supported by substantial neuroimaging evidence.^6^ However, it is still unclear how extensive these changes are and the extent to which they are associated with adult psychopathology.

Previous structural and functional magnetic resonance imaging (fMRI) studies^7,8^ of child abuse have primarily investigated morphologic changes in the brain or used task-based functional imaging to investigate the function of stress-regulatory circuitry. Other studies^9,10^ have explored alterations in resting or intrinsic functional connectivity, allowing for the unrestricted examination of the functional architecture of the brain in absence of task-related biases. A limitation of the current evidence, however, is that most functional connectivity studies^11,12^ of child abuse have focused on the amygdala as a seed and therefore are restricted to identifying alterations in connectivity between the amygdala and other regions of the brain, including the prefrontal cortex, hippocampus, parietal lobe, and striatum. Although this work has been important in understanding how experience of childhood abuse shapes key stress-related brain mechanisms, these approaches have prevented a comprehensive investigation of the functional dynamics of the whole brain (ie, the connectome) with experienced abuse. There is emerging evidence that childhood abuse is associated with more pervasive alterations in functional connectivity. For example, abuse has been associated with altered connectivity between dorsal anterior cingulate cortex and executive control networks,^13^ as well as with increased connectivity among functional systems responsible for an array of processes, including directing attention, external and internal cognition, receiving somatomotor input, and subcortical regions.^14,15^ The generalizability of these initial studies is restricted, however, because they have either focused on short-term impacts of childhood adversity only up to adolescence^14^ or examined functional connectivity alterations only in the context of depression.^15^

The goal of the current study was to address these gaps by using a comprehensive connectome-wide approach to investigate the associations of childhood adversity and alterations in whole-brain intrinsic functional connectivity in adulthood without the restriction of psychiatric disorder. This is an important step forward to disentangle the neural associations of childhood abuse from those of associated psychiatric conditions. Accordingly, we investigated transdiagnostic associations between functional connectivity and reported childhood abuse. On the basis of collective evidence from previous studies,^13,14,15^ we expected that we would find abnormally higher connectivity among the attention, executive control, and sensorimotor networks as well subcortical limbic alterations in connectivity. Our connectomic approach provides a regionally unbiased way to inspect whole-brain connectivity alterations. We also investigated the extent to which abuse-related functional alterations were dependent on abuse occurring before or after adolescence or in association with sex, psychiatric illness, or current symptom severity.

## Methods

### Participants

All procedures for this cohort study were approved by the Western Sydney Area Health Service Human Ethics Committee, and the study followed the Strengthening the Reporting of Observational Studies in Epidemiology (STROBE) reporting guideline. Written informed consent was obtained from all participants. All data were deidentified. There were initially 768 adult participants recruited and tested from January 1, 2009, to December 31, 2015, at the Brain Dynamics Centre at the Westmead Institute for Medical Research, Sydney, Australia. Data analysis was performed from October 1, 2020, to March 31, 2022.

Mental health status was determined by a structured clinical interview (based on the *Diagnostic and Statistical Manual of Mental Disorders* [Fourth Edition]) using the Mini-International Neuropsychiatric Interview, version 5.5.^16^ Participants were recruited through advertisements to participate in clinical research or treatment trials. Exclusion criteria were a history of neurologic disorder, psychosis, or current substance dependence. Participants taking a psychotropic medication were eligible if they were on a stable dosage for at least 2 months before testing. Ethnicity or racial information for participants was not collected; however, the participants were representative of the Sydney population, which is racially diverse.

Self-report measures included the Depression and Anxiety Stress Scale (DASS)^17^ to assess levels of depression, anxiety, and stress and the Early Life Stress Questionnaire^18^ to assess adverse childhood and adolescent events (0-17 years of age) (eMethods in Supplement 1). Participants were asked if they had experienced sexual, physical, or emotional abuse (each separately) and told to select 1 response of 4 choices (none, 0-3, 4-7, 8-12, or 13-17) to indicate the age bracket at which the abuse first occurred. Data for 57 individuals were excluded due to movement on fMRI scans (n = 42) or lack of completion of all fMRI tasks (n = 15). Furthermore, 16 participants were younger than 18 years at the time of completing the study, and 48 participants did not complete the questionnaire, resulting in data from a total of 647 individuals.

Our primary analysis compared all individuals who experienced abuse before the age of 18 years vs those who did not. Because of strong prior evidence of neural and psychological correlates of child abuse being distinctly manifested before adolescence,^19,20^ we also performed a secondary analysis on only those who had experienced abuse before adolescence (before 13 years of age) comparing them with nonabused participants and those abused during adolescence.

### fMRI Acquisition and Preprocessing

Functional MRI data were acquired on a single 3T GE Signa Twinspeed HDxT MR Scanner (GE Healthcare) using an 8-channel phased-array head coil. All participants completed 5 fMRI tasks, of which task-derived intrinsic connectivity data were estimated using a previously validated method.^21,22^ The details of the acquisition, preprocessing, method for task-derived intrinsic connectivity, and handling of motion has been described previously^23^ and in the eMethods in Supplement 1.

### Generation of Functional Brain Connectomes

For each participant, the mean intrinsic time series was extracted from 400 cortical regions and divided into 7 large-scale intrinsic connectivity networks (default mode network [DMN], dorsal attention network [DAN], ventral attention/salience network [VAN], executive control/frontoparietal network [ECN], visual network [VN], somatomotor network [SMN], and limbic network) in addition to an eighth network, which included 36 regions from the subcortex derived from the Brainnetome Atlas.^24^ This time series was correlated pairwise with the time series of every other parcel and the Fisher *z* transformed to create a 436 × 436 interregional functional correlation matrix for each participant.

### Statistical Analysis

The Network-Based Statistic (NBS),^25^ version 1.2 running in MATLAB, version 2018b (MathWorks) was used to perform a 2-sample *t* test for whole-brain connectivity differences between those who experienced abuse before 18 years of age and those who did not, controlling for diagnosis as well as age, sex, years of education, and motion (mean framewise displacement) using an initial threshold of *P* < .001 (Cohen *d* = 0.3) to identify networks of suprathresholded connections followed by permutation testing of these networks at a 2-sided, familywise, Bonferroni-corrected *P* < .05. In supplementary analyses, we also evaluated findings for a range of initial *t* thresholds (eFigure 1 in Supplement 1)

The NBS was also used to compare participants who experienced abuse before 13 years of age with nonabused participants as well as with participants who experienced abuse before and after adolescence, controlling for the same covariates as above. More detailed description of the NBS analysis is in the eMethods in Supplement 1.

For the identified connectome signature, we conducted post hoc analyses in R software, version 4.0.3 (R Foundation for Statistical Computing) (see the eMethods in Supplement 1 for full session information) using a single connectivity metric averaged across all significant connections and for each intrinsic functional network pair that characterized this signature for every individual in both groups. Post hoc analyses of variance tested the association of current clinical diagnosis (depression; anxiety disorders, including general anxiety, social phobia, or panic; stress, including posttraumatic stress disorder (PTSD) and complex PTSD; other, including mild traumatic brain injury; and controls, including healthy and trauma controls), symptom state (DASS), sex, nature of abuse experienced (sexual, emotional, physical, physical and sexual, emotional and sexual, physical and emotional, and all 3), and when abuse was experienced (before or after adolescence as well as different periods within the preadolescence period [0-3, 4-7, or 8-12 years of age]). To control for the number of measures, all post hoc analyses were conducted using a 1-tailed, familywise, Bonferroni-corrected *P* < .05. Because age, sex, years of education, clinical group, and scan motion were different between the abuse and nonabuse groups (Table), we tested associations of these measures with functional connectivity and controlled for them in all post hoc analyses.

**Table.** Participant Characteristics^a^

Characteristic	No. (%)		*P* value for no abuse vs abuse	Abuse at <13 y of age (n = 127)	*P* value for no abuse vs abuse at <13 y of age	Abuse at ≥13 y of age (n = 78)	*P *value for no abuse vs abuse at ≥13 y	*P* value for abuse at <13 y vs ≥13 y	Total (N = 647)
	No abuse (n = 442)	Abuse (n = 205)							
Age, mean (SD), y	32.4 (11.4)	35.1 (12.9)	.01^b^	37.5 (13.3)	<.001^b^	31.1 (11.4)	.35^b^	<.001^b^	33.3 (12.0)
Sex									
Female	212 (48.0)	118 (57.6)	.03^c^	69 (54.3)	.24^c^	49 (62.8)	.02^c^	.29^c^	330 (51.0)
Male	230 (52.0)	87 (42.4)		58 (45.7)		29 (37.2)			317 (49.0)
Years of education, median (range)	16.0 (1.00-18.0)	15.0 (3.00-18.0)	.02^b^	15.0 (3.00-18.0)	.02^b^	15.0 (9.00-18.0)	.25^b^	.35^b^	15.0 (1.00-18.0)
Diagnosis									
General anxiety	6 (1.4)	3 (1.5)	<.001^c^	2 (1.6)	<.001^c^	1 (1.3)	<.001^c^	.14^c^	9 (1.4)
Complicated grief	10 (2.3)	10 (4.9)		7 (5.5)		3 (3.8)			20 (3.1)
Healthy control	195 (44.1)	32 (15.6)		24 (18.9)		8 (10.3)			227 (35.1)
PTSD	22 (5.0)	32 (15.6)		26 (20.5)		6 (7.7)			54 (8.3)
Grief control	21 (4.8)	4 (2.0)		3 (2.4)		1 (1.3)			25 (3.9)
MDD	126 (28.5)	93 (45.4)		47 (37.0)		46 (59.0)			219 (33.8)
mTBI	20 (4.5)	8 (3.9)		5 (3.9)		3 (3.8)			27 (4.2)
Panic disorder	9 (2.0)	8 (3.9)		5 (3.9)		3 (3.8)			18 (2.8)
Social phobia	16 (3.6)	9 (4.4)		5 (3.9)		4 (5.1)			25 (3.9)
Trauma control	17 (3.8)	6 (2.9)		3 (2.4)		3 (3.8)			23 (3.6)
FD (movement on scans), mean (SD)	0.0769 (0.0341)	0.0854 (0.0359)	.005^b^	0.0883 (0.0356)	.002^b^	0.0808 (0.0361)	.38^b^	.15^b^	0.0796 (0.0349)
Movement outliers, mean (SD)	7.41 (8.45)	9.59 (9.43)	.005^b^	10.1 (9.67)	.005^b^	8.81 (9.02)	.21^b^	.34^b^	8.10 (8.82)
Abuse									
Physical									
No	442 (100)	100 (48.8)	NA	55 (43.3)	NA	45 (57.7)	NA	.06^b^	542 (83.8)
Yes	0	105 (51.2)		72 (56.7)		33 (42.3)			105 (16.2)
Sexual									
No	442 (100)	132 (64.4)	NA	72 (56.7)	NA	60 (76.9)	NA	.005^c^	574 (88.7)
Yes	0	73 (35.6)		55 (43.3)		18 (23.1)			73 (11.3)
Emotional									
No	442 (100)	34 (16.6)	NA	23 (18.1)	NA	11 (14.1)	NA	.58^c^	476 (73.6)
Yes	0	171 (83.4)		104 (81.9)		67 (85.9)			171 (26.4)
DASS score, mean (SD)									
Depression	10.6 (11.4)	18.8 (12.4)	<.001^b^	17.5 (12.6)	<.001^b^	20.8 (11.9)	<.001^b^	.07^b^	13.2 (12.3)
Anxiety	5.74 (6.87)	11.0 (8.33)	<.001^b^	11.3 (8.87)	<.001^b^	10.4 (7.41)	<.001^b^	.48^b^	7.40 (7.75)
Stress	11.2 (9.48)	18.1 (10.1)	<.001^b^	18.0 (10.7)	<.001^b^	18.2 (9.17)	<.001^b^	.87^b^	13.4 (10.2)

Abbreviations: DASS, Depression and Anxiety Stress Scale; FD, framewise displacement; MDD, major depressive disorder; mTBI, mild traumatic brain injury; NA, not applicable; PTSD, posttraumatic stress disorder.

^a^ Data are presented as number (percentage) of patients unless otherwise indicated.

^b^ Two-tailed *t* test.

^c^ χ^2^ Test.

## Results

### Participant Demographic Characteristics

A total of 647 individuals (330 female [51.0%] and 317 male [49.0%]; mean [SD] age, 33.3 [12.0]; age, range, 18.2-69.2 years) had usable data for this study. The individuals who had experienced abuse were slightly older (mean difference, 2.7 years; 95% CI, 0.56-4.71; *P* = .01), were more often female (odds ratio [OR], 1.47; 95% CI, 1.05-2.05; *P* = .03), had completed fewer years of education (mean difference, −0.6 years; 95% CI, −0.11 to −1.05; *P* = .02), and were more likely to have a current psychiatric diagnosis (OR, 4.55; 95% CI, 3.07-6.72; *P* < .001), with greater depressive, anxiety, and stress symptoms after controlling for diagnosis (mean difference DASS score, 20.4; 95% CI, 16.1-24.7; *P* < .001). Childhood abuse participants also had greater movement on fMRI scans (mean difference framewise displacement, 0.01; 95% CI, 0.003-0.014; *P* = .003). Motion parameters for the entire data set are provided in eTable 1 in Supplement 1.

The incidence of sexual abuse was greater in those 13 years or older (mean difference, 6.36 years; 95% CI, 2.79-9.93; *P* < .001) compared with those younger than 13 years (OR, 2.54; 95% CI, 1.35-4.79; *P* = .005). Demographic group differences are summarized in the Table.

### Associations of Childhood Abuse With the Functional Connectome in Adults

**Figure 1.**
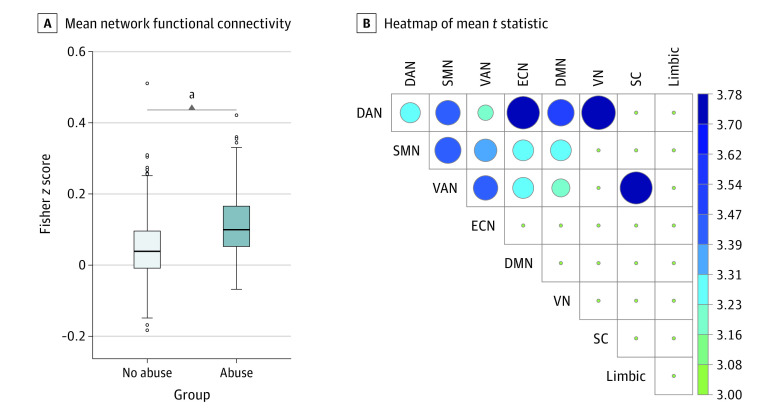
Network of Connections Increased in Individuals Exposed to Childhood Abuse A total of 261 edges and 117 nodes had significantly higher functional connectivity in the abuse group compared with the no abuse group (familywise, Bonferroni-corrected *P* = .01). A, Difference in mean functional connectivity (Fisher *z* transformed correlation coefficient of neural connectivity) of all significant interregional connections compared between groups. The boxes represent the IQR (first quartile [Q1] to third quartile [Q3]), center lines represent the median, and whiskers represent variability (Q1/Q3 ± 1.5*IQR). B, Heatmap of the mean *t* statistic of significantly different connections within and between the 8 primary functional networks (dorsal attention network [DAN], somatomotor network [SMN], ventral attention/salience network [VAN], executive control/frontoparietal network [ECN], default mode network [DMN], visual network [VN], subcortical [SC] region, and limbic network). Larger circle size and darker color represent a greater mean *t* statistic of connections (larger difference in connectivity between the 2 groups). Networks are ordered based on their overall contribution to the difference in connectivity. ^a^*P* < .01

No significant differences in functional connectivity were found between those who had experienced abuse before 18 years of age vs those who did not. However, for individuals who had experienced abuse before 13 years of age only (preadolescence) vs those with no abuse, a significant network of higher connectivity was identified (mean [SD], 0.12 [0.09] vs 0.05 [0.08]; mean difference, 0.07; 95% CI, 0.05-0.08; familywise, Bonferroni-corrected *P* = .01; Cohen *d* = 0.82) (Figure 1A), comprising 261 edges and 117 nodes (eFigure 2 in Supplement 1). This network was dominated by alterations in connectivity: (1) within and between DAN, SMN, and VAN; (2) between these 3 networks and the ECN and DMN; (3) between DAN and VN; and (4) between the putamen (subcortex) and VAN (Figure 1B). The summary of edges from each within or between intrinsic connectivity network pair and correlations of connectivity with demographic measures are shown in the eAppendix, eFigures 3 and 4, and eTables 2 and 3 in Supplement 1.

### Association of Connectivity With Diagnosis and Current Symptoms 

**Figure 2.**
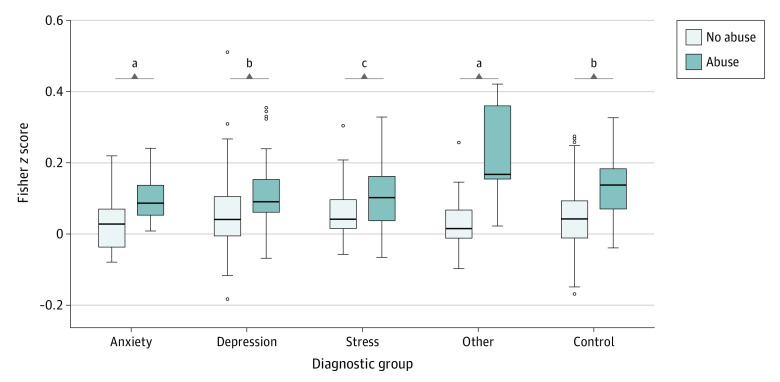
Mean Network Functional Connectivity by Diagnostic Group A significant interaction effect was found for abuse × diagnostic category and mean functional connectivity (*P* = .049), with connectivity differences between the abuse and no abuse groups significant for the depression, anxiety, and control groups but not the stress disorder group. No connectivity differences were observed between the diagnostic categories. The boxes represent the IQR (first quartile [Q1] to third quartile [Q3]), center lines represent the median, and whiskers represent variability (Q1/Q3 ± 1.5*IQR). ^a^*P* < .05. ^b^*P* < .01. ^c^*P* < .001.

There was a significant abuse × diagnostic category interaction for mean connectivity of the identified connectome signature. In post hoc analyses, connectivity differences were significant for the no abuse vs abuse groups for the depression (mean [SD], 0.05 [0.09] vs 0.11 [0.09]; *P* < .001; Cohen *d* = 0.66), anxiety (mean [SD], 0.03 [0.08] vs 0.10 [0.08]; *P* = .02; Cohen *d* = 0.87), and control (mean [SD], 0.04 [0.08] vs 0.13 [0.09]; *P* < .001; Cohen *d* = 1.1) groups but not in the stress disorder group (mean [SD], 0.06 [0.09] vs 0.11 [0.10]; *P* = .10; Cohen *d* = 0.52) (Figure 2). However, this interaction was not significant for any of the individual internetwork connectivity pairs after correcting for multiple comparisons. There were also no connectivity differences among the diagnostic categories (main effect of diagnosis). Current symptoms of depression, anxiety, and stress (DASS scores) had no significant association with functional connectivity (eAppendix and eFigure 5 in Supplement 1).

### Association of Connectivity to Sex

**Figure 3.**
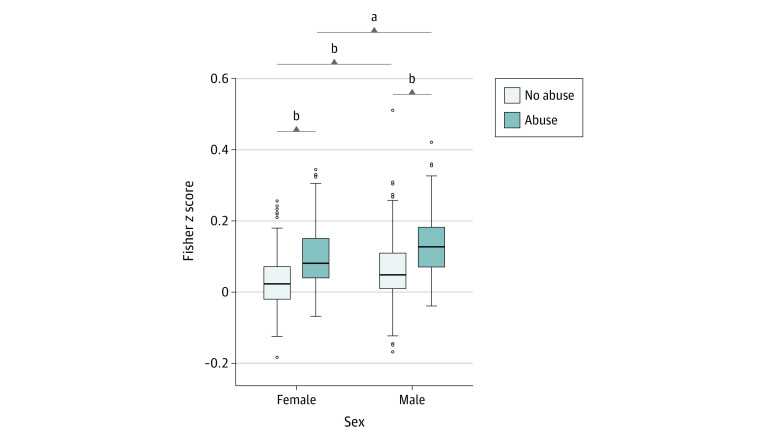
Mean Functional Connectivity of Identified Network in the Abuse and No Abuse Groups by Sex Overall, men had significantly higher mean functional connectivity of the network compared with women (*P* < .001, F = 1.536, controlling for clinical group, mean framewise displacement, age, and years of education). However, there was no interaction between abuse and sex. The boxes represent the IQR (first quartile [Q1] to third quartile [Q3]), center lines represent the median, and whiskers represent variability (Q1/Q3 ± 1.5*IQR). ^a^*P* < .05. ^b^*P* < .001.

Men had significantly higher mean network connectivity within the identified connectome signature compared with women (mean difference, 0.02; 95% CI, 0.01-0.04; *P* < .001; Cohen *d* = 0.31). However, there were no abuse × sex interactions, suggesting that connectivity differences between the abuse groups were not different between men and women (Figure 3).

### Differences in Connectivity Based on Differences in Age at Abuse Onset, Type, and Load

**Figure 4.**
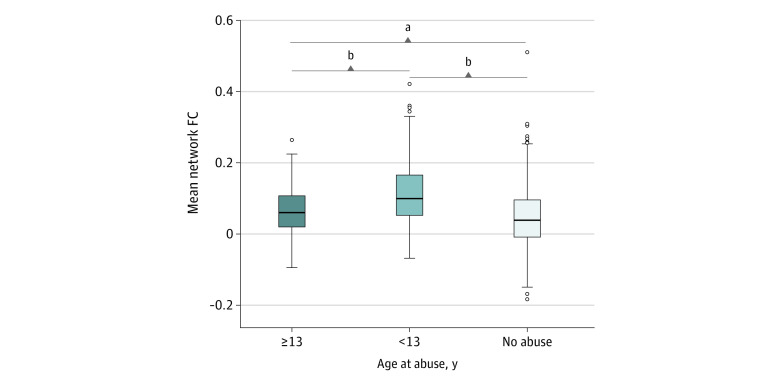
Post Hoc Analysis Comparing Mean Functional Connectivity (FC) of Identified Network in Participants Abused in Adolescence (≥13 Years of Age) With Connectivity of Abused Participants in Childhood (<13 Years of Age) Those abused at 13 years or older had significantly lower functional connectivity compared with those abused before the age of 13 years but had comparable connectivity to individuals who never experienced abuse. The boxes represent the IQR (first quartile [Q1] to third quartile [Q3]), center lines represent the median, and whiskers represent variability (Q1/Q3 ± 1.5*IQR). ^a^*P* = .36. ^b^*P* < .001.

There were significant differences in functional connectivity between individuals who experienced abuse before vs during adolescence (mean difference, 0.06; 95% CI, 0.04-0.08; *P* < .001; Cohen *d* = 0.68), even when controlling for other nonimaging cohort differences (age, clinical group, and sexual abuse) (Figure 4). This difference was significant between DMN and DAN (mean difference, 0.07; 95% CI, 0.03-0.10; *P* < .001), ECN and SMN (mean difference, 0.06; 95% CI, 0.03-0.09; *P* < .001), VAN and SMN (mean difference, 0.07; 95% CI, 0.02-0.10; *P* < .001), DMN and SMN (mean difference, 0.05; 95% CI, 0.02-0.08; *P* = .001), DAN and SMN (mean difference, 0.06; 95% CI, 0.02-0.10; *P* = .001), and ECN and DAN (mean difference, 0.08; 95% CI, 0.03-0.14; *P* = .005) (eFigure 6 in Supplement 1) after controlling for multiple comparisons. There were no significant differences in connectivity based on age when first experiencing abuse when using groupings before the age of 13 years (0-3, 4-7, and 8-12 years), number of abuse types experienced, or difference between abuse type (eFigure 7 in Supplement 1). Different abuse types for each age band are provided in eTable 4 in Supplement 1.

## Discussion

This study aimed to investigate a potential transdiagnostic whole-brain functional connectome signature associated with early experience of abuse. We found a network of connections with abnormally higher intrinsic functional connectivity, particularly in individuals who experienced abuse before adolescence. Connectivity within this network was also significantly higher compared with those who experienced abuse in adolescence, adding to the body of existing evidence that timing of experienced abuse has distinct neural manifestations.^26,27,28^ Age when abuse is experienced is critical in moderating the risk and type of psychopathology, with individuals during the preschool and preadolescent periods especially sensitive for increased risk of depression,^29^ dissociative symptoms,^30^ and suicidal ideations.^19^

This connectivity signature was primarily characterized by greater connectivity in connections within the VAN, DAN, and SMN and among these 3 networks with the ECN and DMN of the brain. This pattern supports a prior study^15^ of depressed individuals that found a history of child abuse was associated with altered connectivity of dorsal attention to the SMN, VAN, and ECN, as well as within network connectivity of DAN. The current finding also accords with evidence that childhood maltreatment is associated with increased connectivity among the ECN, DAN, and DMN.^14^ The convergent evidence that childhood abuse is associated with alterations in networks related to attention, cognitive control, and somatomotor systems may reflect heightened awareness of one’s environment due to being exposed to high levels of threat at an early age.^31^ This interpretation is supported by the evidence of early abuse being associated with greater connectivity of amygdala-related networks,^11,12^ as well as vigilance to potential threats as measured by emotional Stroop^32^ and facial encoding^33^ paradigms. Although hypervigilance to threat is typically considered indicative of anxiety disorders,^34^ this pattern may suggest that adults who have experienced childhood abuse may also have a preferential sensitivity to environmental cues.

This study is the first, to our knowledge, to evaluate the associations of child abuse on the functional brain connectome of adults in a large transdiagnostic sample. Although a much higher proportion of individuals with psychiatric diagnoses had a history of child abuse than those who did not, connectivity differences associated with child abuse remained significant when controlling for diagnosis and current symptom states. This finding could suggest that the identified functional connectome signature is likely representative of independent effects of child abuse and not confounded by current diagnosis or symptom severity. It is possible that the functional connectome signature observed in this study represents a vulnerability factor that in combination with other environmental, genetic, and psychological factors may contribute to risk of a psychiatric disorder.^4^ This theory may also explain why we did not observe a significant effect of diagnosis on connectivity in this signature (despite including controls as a group). We observed reduced connectivity differences between abused and nonabused participants in stress disorders relative to depression, anxiety disorders, and healthy individuals. This observation could be attributed to well-documented overlap in stress disorder symptoms (eg, hypervigilance to threat cues) and experience of childhood abuse.^35,36^

We also investigated sex differences. Previous work,^14,37^ particularly in adolescents, has indicated that the neural impacts of childhood abuse may be moderated by sex. Although more women than men had experienced childhood abuse in our cohort, there were no differential connectivity associations based on sex between individuals who had and hadn’t experienced abuse. However, we did observe overall sex differences, with men demonstrating greater connectivity than women. There is considerable evidence that neuronal development occurs at different rates in men and women,^38^ particularly in identified networks,^39^ which may contribute to men being more susceptible to the effect of child maltreatment on hyperconnectivity.

In our study, no connectivity alterations were observed in amygdala, hippocampal or limbic, or subcortical regions, except for 2 connections between the putamen to right and left sides of the VAN. The previous 2 whole-brain studies^14,15^ of functional connectivity in those with child abuse histories also found no or minimal connectivity differences involving limbic or subcortical regions. Notably, seed-based analytical approaches that have evaluated amygdala and hippocampal functional connectivity have found alterations associated with childhood trauma and maltreatment.^11,12^ This pattern could suggest differences due to variable methodologic approaches. Although seed-based studies bias findings toward the seed of choice or due to the use of task stimuli that might expose unique functional deficits and their associated neural alterations, the use of a whole-brain parcellation may also bias findings toward the largest areas of the brain and those with the greatest numbers of connections (ie, neocortical regions). Nevertheless, the finding of altered connectivity related to the putamen and VAN is unsurprising given known reports of childhood adversities associated with the development of blunted responses to reward cues in adulthood.^40^

### Limitations

Our retrospective measures of childhood maltreatment may not have been sufficiently sensitive to identify detailed histories of the nature and extent of the abuse. Previous studies have indicated differential patterns of connectivity depending on abuse type,^6,15^ amount of childhood trauma, and association with symptom severity.^13,14^ However, we did not observe any such associations, which could be due to small numbers in subgroups measuring these factors and/or lack of detail in these measures. It is possible that more detailed measurement of abuse with a measure, such as the Maltreatment and Abuse Chronology of Exposure Scale,^41^ may have allowed us to interrogate our data in terms of more nuanced aspects of the timing and nature of adverse childhood experiences. In this context, we note that our measure did not assess childhood neglect, which is associated with distinct patterns of brain changes.^14,20,42^ In addition, we did not administer a continuous measure of PTSD, a common psychiatric disorder after childhood abuse, which may have provided a more sensitive measure of how the functional connectome may map onto the severity of PTSD symptoms.

## Conclusions

This study provides evidence for whole-brain alterations to intrinsic functional connectivity in individuals with a history of early experience of abuse that appears to be transdiagnostic. These alterations are characterized by higher connectivity through the SMN, DAN, and VAN and between these networks and the ECN and DMN. The pattern of alterations may reflect impaired attentional and perceptual processing that could result from the stress of early-life adversity. These factors may subsequently interact with other factors, such as genetic or environmental processes, that may increase the risk of subsequent psychiatric disorders.

## Notes

Supplement 1. **eMethods.** Supplemental Methods **eFigure 1.** Observed Change in NBS Network Associated With Child Abuse at Different T-Thresholds **eFigure 2.** Visualisation of Significant Nodes and Edges Identified in NBS as Greater in Participants That Experiences Abuse **eFigure 3.** Differences in Intrinsic Functional Connectivity Network Pairs That Comprised the Functional Connectome Differentiating Abused and Non-Abused Individuals **eFigure 4.** Correlation of Total Mean Network Connectivity to Demographic Characteristics **eFigure 5.** Correlation of Network Mean Connectivity With Clinical Measures **eFigure 6.** Differences in Mean Connectivity of Individual Intrinsic Connectivity Networks Between Those Abused Before vs After the Age of 13 **eFigure 7.** Differences in Mean Connectivity Based on Abuse Type, Load and Onset Before the Age of 13 **eAppendix.** Supplemental Results **eTable 1.** Summary of Motion Parameters for Entire Dataset **eTable 2.** Summary of Network-wise Distribution of Edges, Difference Between Groups **eTable 3.** List of All Significant Connections Identified in NBS as Increased in Participants That Experienced Child Abuse **eTable 4.** Proportion of Abuse Types at Different Ages of Onset
eReferences

Supplement 2.
Data Sharing Statement
