# Supplementary material for: Association of Neural Connectome With Early Experiences of Abuse in Adults
Source: JAMA Netw Open. 2023 Jan 26;6(1):e2253082. doi: 10.1001/jamanetworkopen.2022.53082 (PMC9880798; doi:10.1001/jamanetworkopen.2022.53082)
Supplement: Supplement 1. — eMethods. Supplemental Methods eFigure 1. Observed Change in NBS Network Associated With Child Abuse at Different T-Thresholds eFigure 2. Visualisation of Significant Nodes and Edges Identified in NBS as Greater in Participants That Experiences Abuse eFigure 3. Differences in Intrinsic Functional Connectivity Network Pairs That Comprised the Functional Connectome Differentiating Abused and Non-Abused Individuals eFigure 4. Correlation of Total Mean Network Connectivity to Demographic Characteristics eFigure 5. Correlation of Network Mean Connectivity With Clinical Measures eFigure 6. Differences in Mean Connectivity of Individual Intrinsic Connectivity Networks Between Those Abused Before vs After the Age of 13 eFigure 7. Differences in Mean Connectivity Based on Abuse Type, Load and Onset Before the Age of 13 eAppendix. Supplemental Results eTable 1. Summary of Motion Parameters for Entire Dataset eTable 2. Summary of Network-wise Distribution of Edges, Difference Between Groups eTable 3. List of All Significant Connections Identified in NBS as Increased in Participants That Experienced Child Abuse eTable 4. Proportion of Abuse Types at Different Ages of Onset eReferences [file jamanetwopen-e2253082-s001.pdf]

Supplementary Online Content

Korgaonkar MS, Breukelaar IA, Felmingham K, Williams LM, Bryant RA. Association of neural connectome with early experiences of abuse in adults. *JAMA Netw Open*. 2023;6(1):e2253082. doi:10.1001/jamanetworkopen.2022.53082

eMethods. Supplemental Methods

eFigure 1. Observed Change in NBS Network Associated With Child Abuse at Different T-Thresholds

eFigure 2. Visualisation of Significant Nodes and Edges Identified in NBS as Greater in Participants That Experiences Abuse

eFigure 3. Differences in Intrinsic Functional Connectivity Network Pairs That Comprised the Functional Connectome Differentiating Abused and Non-Abused Individuals

eFigure 4. Correlation of Total Mean Network Connectivity to Demographic Characteristics

eFigure 5. Correlation of Network Mean Connectivity With Clinical Measures

eFigure 6. Differences in Mean Connectivity of Individual Intrinsic Connectivity Networks Between Those Abused Before vs After the Age of 13

eFigure 7. Differences in Mean Connectivity Based on Abuse Type, Load and Onset Before the Age of 13

eAppendix. Supplemental Results

eTable 1. Summary of Motion Parameters for Entire Dataset

eTable 2. Summary of Network-wise Distribution of Edges, Difference Between Groups

eTable 3. List of All Significant Connections Identified in NBS as Increased in Participants That Experienced Child Abuse

eTable 4. Proportion of Abuse Types at Different Ages of Onset

eReferences

This supplementary material has been provided by the authors to give readers additional information about their work.

eMethods. Supplemental Material

Early Life Questionnaire (ELSQ)

Exposure to early life stressors between 0 and 17 years of age was assessed using the Early Life Stress Questionnaire (ELSQ)[1]. The ELSQ is scored dichotomously for the presence/absence of exposure to 18 specific early life stressors events reported in previous studies to be traumatic or extremely stressful in childhood.

This scale has been validated to capture equivalent prevalence rates to exposures to early life stressors as done by previous large international studies on early life stress reporting and has been established in a large community sample that higher reporting of abuse on this scale to be predictive of high clinical symptoms[2]. The scale has also found to be comparative to Criterion A evaluation of trauma criteria for PTSD and has been shown to differentially predict higher self-reported depressive and anxiety symptoms scores as compared to adult trauma exposure.

fMRI acquisition

Functional MRI data was acquired using an echo planar imaging sequence with the following parameters: repetition time (TR)=2,500ms, echo time (TE)=27.5ms, matrix=64x64, field of view (FOV)=24cm, flip angle=90°, 120 volumes with a total scan time of 5min and 8s. Forty slices, each 3.5mm thick, covered the whole brain in each volume. Three dummy scans were also acquired at the start of every acquisition to allow magnetization to stabilize to steady state. T1-weighted images were acquired in the sagittal plane using a 3D SPGR sequence (FOV=256mm, TR=8.3ms; TE=3.2ms; flip angle=11°; TI=500ms; NEX=1 and ASSET=1.5; frequency direction: S/I; 256x256 matrix, 180 contiguous slices, 1mm isotropic voxels) and used for normalization of the functional data.

fMRI pre-processing

All pre-processing was performed on a linux system running Centos 7.0 using in-house Matlab (2018b) batch scripts which ran realignment and normalization through FSL 5.0 and all other steps through spm8. The functional MRI tasks involved the Go-NoGo (response inhibition), auditory oddball, working memory (n-back), facial emotion processing and emotional reappraisal. For each of the five fMRI tasks, the blood oxygen level dependent (BOLD) responses for each experimental condition were modelled in the general linear model framework. The mean signal time course derived from the cerebral spinal fluid (CSF) and white matter masks, as well as the temporal masks for movement outliers (below), were also included as covariates in the model to remove physiological noise. The variance in BOLD signal associated with each of the stimuli in the task was then modelled as a covariate and the remaining residual images represented the task-derived resting state signal. After this, a band-pass filter (0.009 Hz $< f < 0.08$ Hz) was applied. This process results in a movement and task-effect-corrected time series of 600 volumes (120 volumes x 5) from which intrinsic-resting state connectivity (correlation of change in BOLD signal between voxels/nodes across time) can be measured. While this "intrinsic connectivity" is different from pure resting-state connectivity previous studies have shown that it exhibits a very similar functional connectivity pattern reflecting the inherent connectivity of the large-scale networks in the brain[3,4].

Movement outliers were identified as framewise displacement (FD) of 0.3mm or greater or a difference in scaled signal intensity greater than 10 from one volume to the next. The two volumes before and one after each outlier were also included[5-8]. The Volterra expansion of

© 2023 Korgaonkar MS et al. *JAMA Network Open.*

twenty-four realignment parameters was also modelled for each task[9]. Participants with a total mean framewise displacement FD of greater than 0.2 were excluded.

© 2023 Korgaonkar MS et al. *JAMA Network Open.*

Network Based Statistics Analysis

The NBS is a validated non-parametric statistical approach that addresses the multiple comparison problem by testing the null hypothesis based on interconnected subnetworks rather than individual connections. Briefly, group differences were assessed using a two-sample t-test and connections that met significance above a t-statistic threshold of 3.3 (equivalent to $p<0.001$ significance level) was used to form a network, or component, of supra-thresholded connections. Statistical significance of each component was then tested relative to components formed through random permutation to obtain correction at familywise error $p<0.05$. For significant findings, supplementary NBS analyses were also run at t-statistic threshold of 3.1, 3.5, 3.7, 3.9 and 4.1 to examine the relative strength vs breadth of the network identified and are shown in eFigure 1.

© 2023 Korgaonkar MS et al. *JAMA Network Open.*

Post hoc analysis – R session information

```
R version 4.0.3 (2020-10-10)
Platform: x86_64-apple-darwin17.0 (64-bit)
Running under: macOS  12.6

Matrix products: default
LAPACK: /Library/Frameworks/R.framework/Versions/4.0/Resources/lib/libRlapack.dylib

locale:
[1] en_AU.UTF-8/en_AU.UTF-8/en_AU.UTF-8/C/en_AU.UTF-8/en_AU.UTF-8

attached base packages:
[1] stats     graphics  grDevices utils     datasets  methods   base

other attached packages:
 [1] ggpubr_0.4.0     broom_0.7.12     caret_6.0-91     descr_1.1.5      MASS_7.3-55
dlookr_0.5.4     webshot_0.5.2
 [8] magick_2.7.3     MatchIt_4.3.4    table1_1.4.2     Hmisc_4.6-0      Formula_1.2-4
survival_3.3-1   lattice_0.20-45
[15] boot_1.3-28      kableExtra_1.3.4 readxl_1.3.1     forcats_0.5.1    stringr_1.4.0
dplyr_1.0.8      purrr_0.3.4
[22] readr_2.1.2      tidyr_1.2.0      tibble_3.1.6     ggplot2_3.3.5
tidyverse_1.3.1  plyr_1.8.6

loaded via a namespace (and not attached):
  [1] backports_1.4.1      systemfonts_1.0.4    splines_4.0.3        listenv_0.8.0
digest_0.6.29
  [6] foreach_1.5.2        htmltools_0.5.2      fansi_1.0.2          magrittr_2.0.2
checkmate_2.0.0
 [11] cluster_2.1.2        tzdb_0.2.0           recipes_0.2.0        globals_0.14.0
modelr_0.1.8
 [16] gower_1.0.0          extrafont_0.17       sysfonts_0.8.8       extrafontdb_1.0
svglite_2.1.0
 [21] hardhat_0.2.0        jpeg_0.1-9           colorspace_2.0-3     rvest_1.0.2
haven_2.4.3
 [26] xfun_0.30            crayon_1.5.0         jsonlite_1.8.0       libcoin_1.0-9
iterators_1.0.14
 [31] glue_1.6.2           gtable_0.3.0         ipred_0.9-12         car_3.0-12
Rttf2pt1_1.3.10
 [36] future.apply_1.8.1   abind_1.4-5          scales_1.1.1         mvtnorm_1.1-3
DBI_1.1.2
 [41] rstatix_0.7.0        Rcpp_1.0.8.3         showtextdb_3.0       viridisLite_0.4.0
xtable_1.8-4
 [46] htmlTable_2.4.0      foreign_0.8-82       stats4_4.0.3         lava_1.6.10
prodlim_2019.11.13
 [51] htmlwidgets_1.5.4    httr_1.4.2           RColorBrewer_1.1-2   ellipsis_0.3.2
pkgconfig_2.0.3
 [56] nnet_7.3-17          dbplyr_2.1.1         utf8_1.2.2           tidyselect_1.1.2
rlang_1.0.2
 [61] reshape2_1.4.4       later_1.3.0          munsell_0.5.0        cellranger_1.1.0
tools_4.0.3
 [66] cli_3.2.0            generics_0.1.2       evaluate_0.15        fastmap_1.1.0
yaml_2.3.5
 [71] ModelMetrics_1.2.2.2 knitr_1.37           fs_1.5.2             showtext_0.9-5
future_1.24.0
 [76] nlme_3.1-155         reactable_0.2.3      mime_0.12            xml2_1.3.3
compiler_4.0.3
 [81] rstudioapi_0.13      curl_4.3.2           png_0.1-7            ggsignif_0.6.3
pagedown_0.17
 [86] reprex_2.0.1         stringi_1.7.6        gdtools_0.2.4        hrbrthemes_0.8.0
Matrix_1.4-0
```

© 2023 Korgaonkar MS et al. *JAMA Network Open.*

```
 [91] vctrs_0.3.8          pillar_1.7.0         lifecycle_1.0.1      data.table_1.14.2
httpuv_1.6.5
 [96] R6_2.5.1             latticeExtra_0.6-29  promises_1.2.0.1     gridExtra_2.3
parallelly_1.30.0
[101] codetools_0.2-18     assertthat_0.2.1     withr_2.5.0          parallel_4.0.3
hms_1.1.1
[106] grid_4.0.3           rpart_4.1.16         timeDate_3043.102    class_7.3-20
rmarkdown_2.16
[111] inum_1.0-4           carData_3.0-5        pROC_1.18.0          partykit_1.2-15
shiny_1.7.1
[116] lubridate_1.8.0      base64enc_0.1-3
```

© 2023 Korgaonkar MS et al. *JAMA Network Open.*

eFigure 1. Observed change in NBS network associated with child abuse at different t-thresholds. Impact of different t-statistic threshold on strength and breadth of network differences between abuse and non-abused participants.

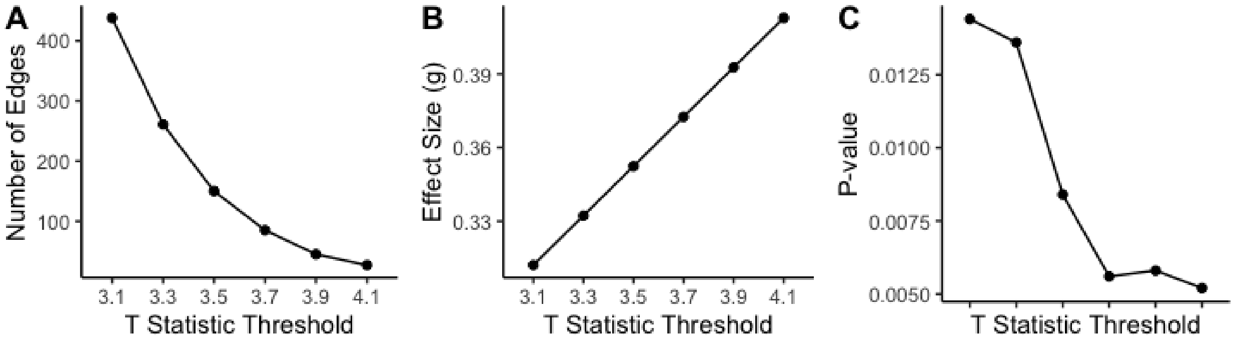

© 2023 Korgaonkar MS et al. *JAMA Network Open.*

eFigure 2. Visualisation of significant nodes and edges identified in NBS as greater in participants that experiences abuse. All 117 significant nodes and 261 edges of the network that had higher connectivity in abused participants relative to non-abused participants mapped onto the surface of the brain using BrainNet viewer. Nodes are colour coded based on the intrinsic connectivity network they belong too.

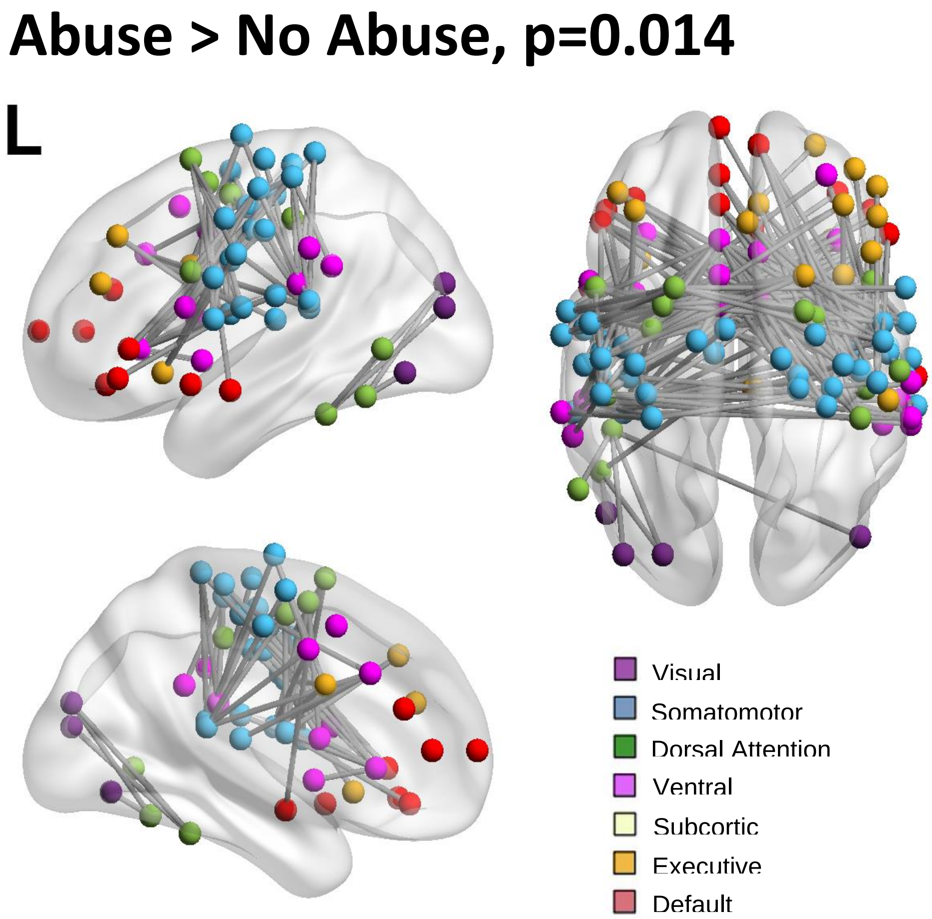

© 2023 Korgaonkar MS et al. *JAMA Network Open.*

eFigure3. Differences in intrinsic functional connectivity network pairs that comprised the functional connectome differentiating abused and non-abused individuals. *, **, ***, **** indicated significates level at p<0.05, 0.01. 0.001, 0.0001 respectively.

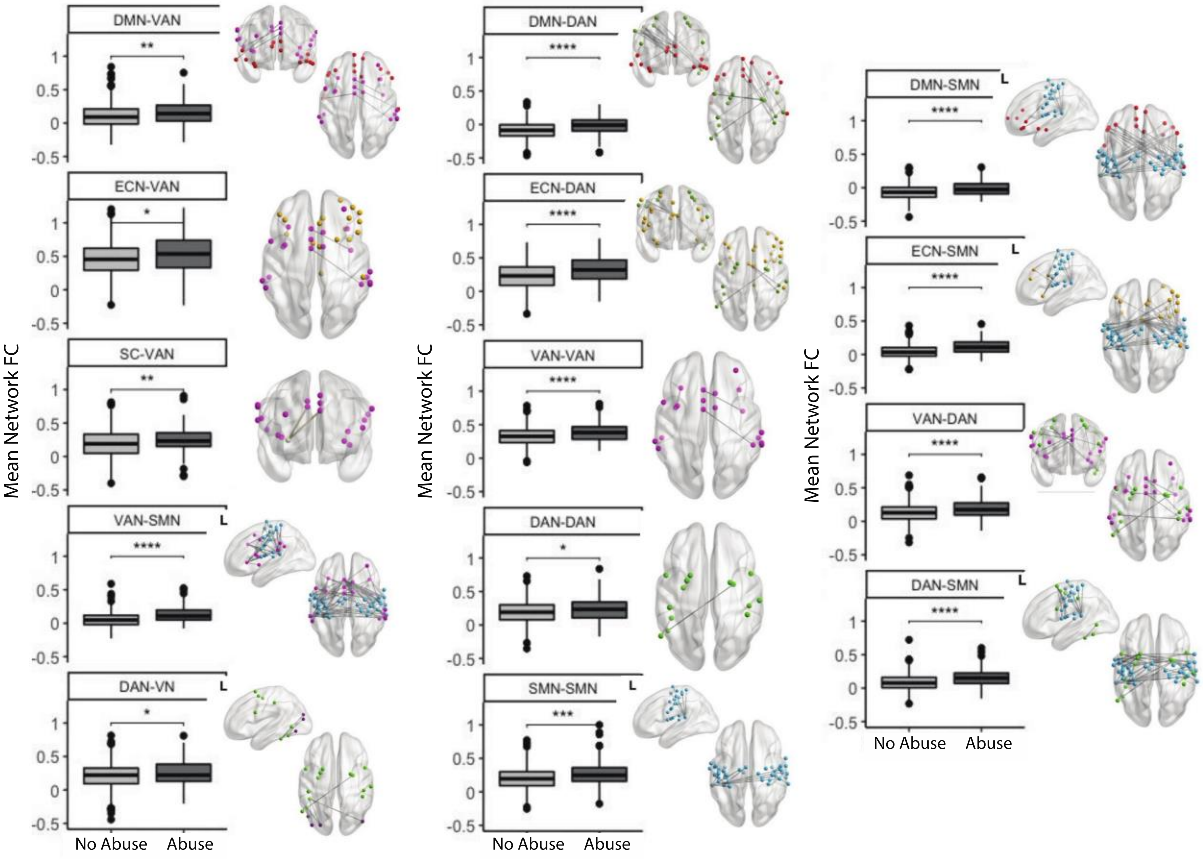

© 2023 Korgaonkar MS et al. *JAMA Network Open.*

eFigure 4. Correlation of Total Mean Network Connectivity to Demographic Characteristics. Age is not significantly associated with mean total network connectivity, when controlling for clinical group, gender, YOE and meanFD. YOE is significantly associated with mean total network connectivity, when controlling for clinical group, gender, age and mean FD (p=0.0372, t=-2.088). Mean FD is significantly associated with mean total network connectivity, when controlling for clinical group, gender, age, and mean FD (p =0.0016, t=3.172).

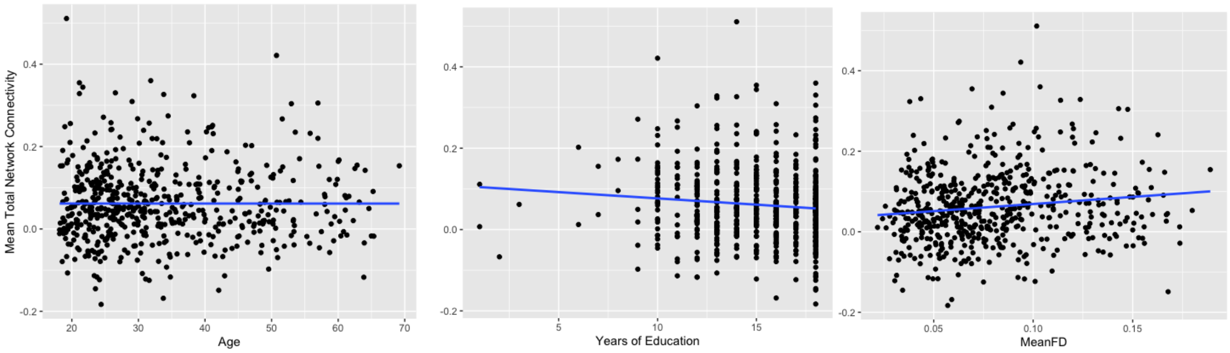

© 2023 Korgaonkar MS et al. *JAMA Network Open.*

eFigure 5. Correlation of network mean connectivity with clinical measures. No significant association with a) depression or b) anxiety score or c) stress score after controlling for clinical group, gender, age, YOE, and mean FD within the abuse group only.

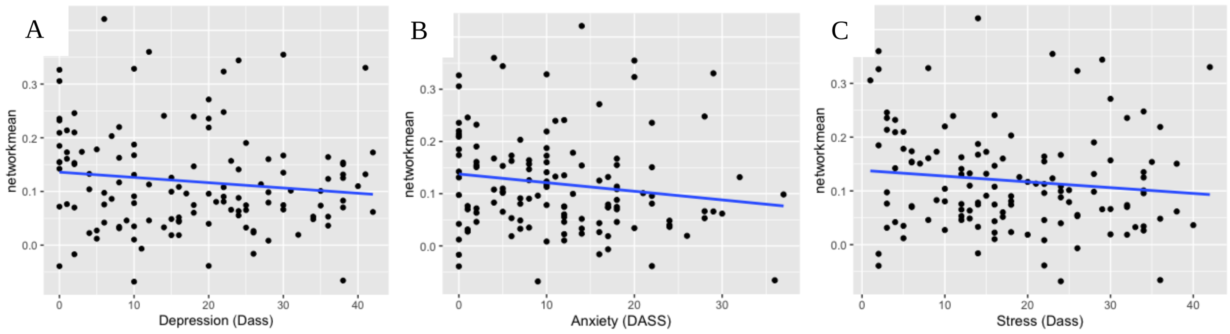

© 2023 Korgaonkar MS et al. *JAMA Network Open.*

eFigure 6. Differences in mean connectivity of individual intrinsic connectivity networks between those abused before vs after the age of 13. Differences in specific connections are marked with *, p<0.05, ** p<0.01, ***p<0.001 correcting for multiple comparisons. Abbreviations: FC- Functional Connectivity, DMN - Default mode network, DAN - dorsal attention network, ECN - executive control/frontoparietal network, SMN - somatomotor network, SC - subcortical, VAN - ventral attention/salience network, VN - visual network.

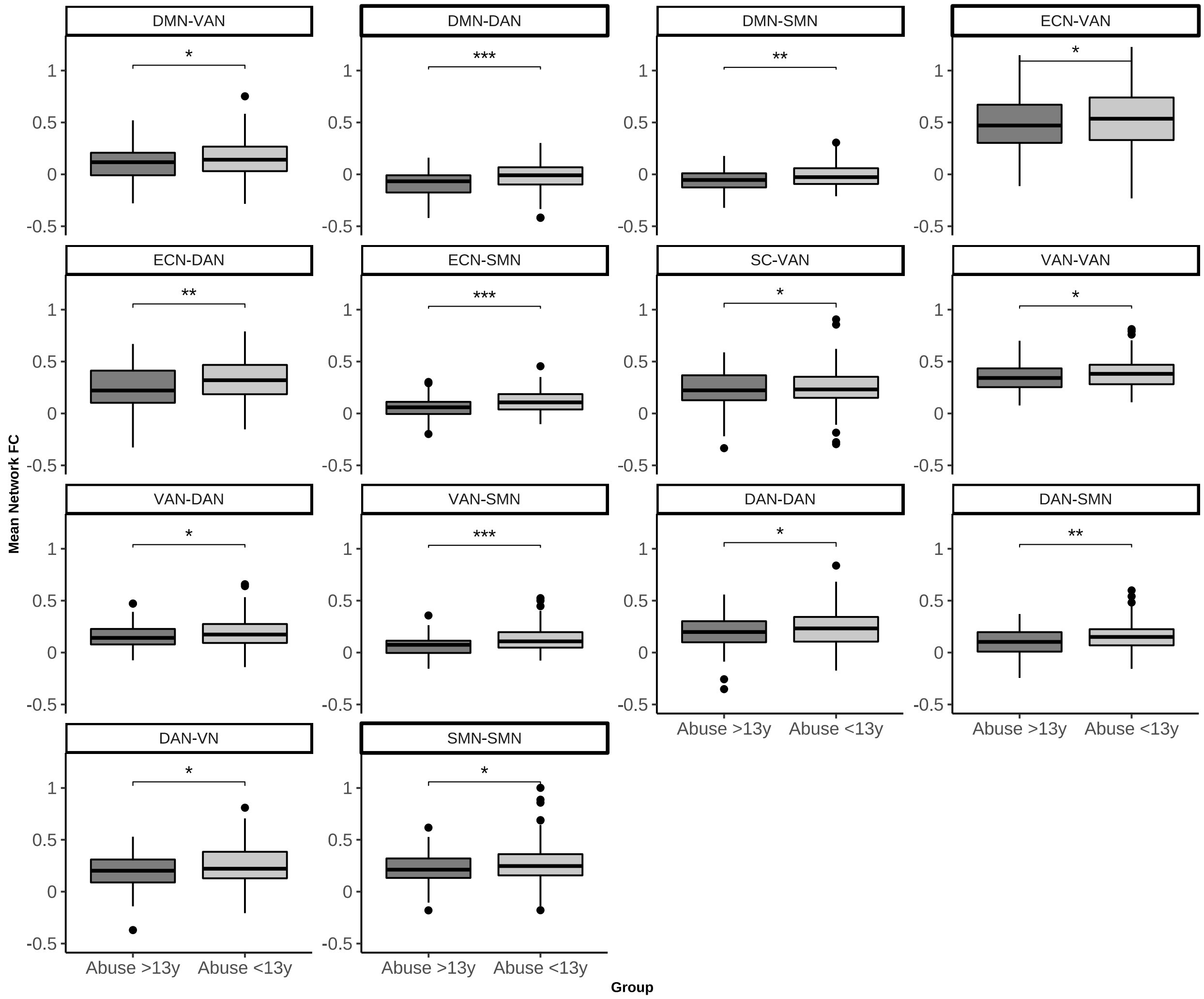

© 2023 Korgaonkar MS et al. *JAMA Network Open.*

eFigure 7. Differences in mean connectivity based on abuse type, load and onset before the age of 13. A. Distribution of abuse load, B. Abuse Load by total mean network connectivity. No significant difference in total mean network connectivity depending on number of abuse types experience (ANOVA controlling for gender, diagnosis, age, mean framewise displacement, years of education – not significant) C. Distribution of abuse types experiences, D. Mean Network Connectivity by abuse type No significant difference were observed in total mean network connectivity between abuse types.

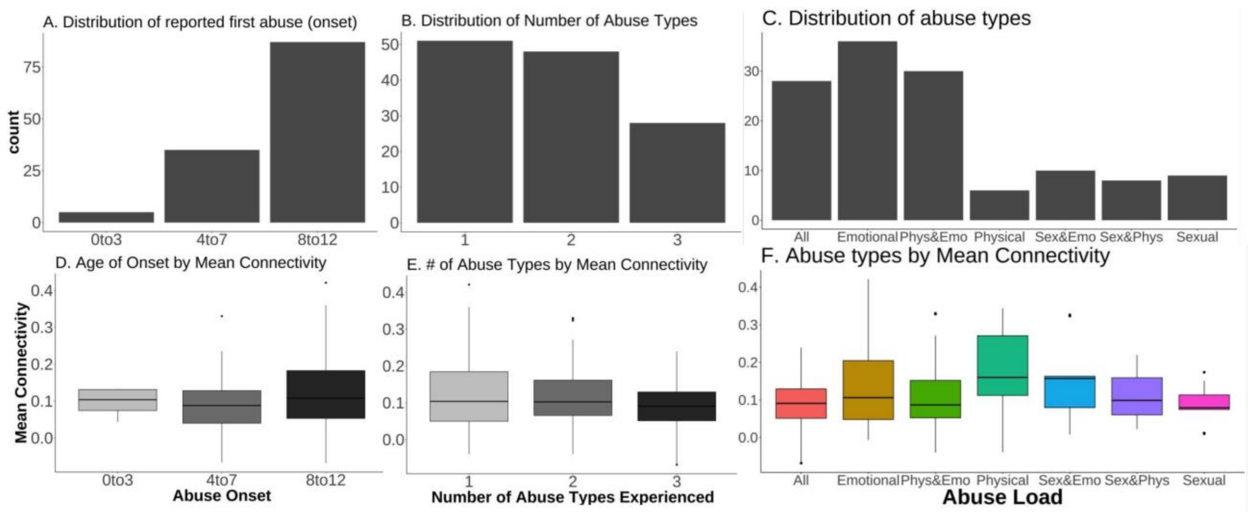

© 2023 Korgaonkar MS et al. *JAMA Network Open.*

eAppendix. Supplemental Results

NBS Analyses at different initial component forming t thresholds

Supplementary NBS analyses were also run at t-statistic threshold of 3.1, 3.5, 3.7, 3.9 and 4.1 to examine the relative strength vs breadth of the network identified. The presence of the network at lower threshold indicates that the network is weak but extensive, while the present of the network at higher threshold indicates that the network is strong but more focal (eFigure 1).

Association of connectivity in the abuse signature with demographic measures

We controlled for age, gender, years of education, clinical diagnosis, and scan motions in our main NBS analysis to identify the connectome signature of abuse. To evaluate how connectivity in this signature was associated with these controlled measures, we conducted post-hoc correlation analyses with each of these measures and mean functional connectivity of the signature. We found that age was not significantly associated with mean total network connectivity ($p=0.93$, $r=0.09$) but that there was a significant correlation of mean total network connectivity with years of education ($p=0.0372$, $t=-2.088$) and scan motion i.e. mean framewise displacement (FD) ($p =0.0016$, $t=3.172$) (eFigure 3). Mean connectivity was also significantly higher in in males ($p<0.001$, $F = 1.536$) than females, after controlling for diagnostic group, mean FD, age and years of education.

Association of connectivity in the abuse signature with clinical measures

Connectivity differences remained significant between abuse and non-abuse groups additionally controlling for current symptoms of depression, anxiety, and stress (DASS scores) both when these variables were included in NBS between group comparison and post-hoc (using mean connectivity of total network and of each inter-network pair; $pFWE<0.05$). There were also no

© 2023 Korgaonkar MS et al. *JAMA Network Open.*

significant associations between connectivity and these symptom measures within the abuse group (eFigure 4). We also investigated the correlation between depression, anxiety and stress scores using the DASS and mean network connectivity, controlling for clinical group, gender, age, YOE and mean FD.

NBS analysis of connectivity depending on onset of abuse in childhood vs adolescence

Whole connectome analyses with NBS, controlling for diagnosis, gender, age, years of education and motion to compare connectivity was performed in the cohort of individuals who experienced abuse over the age of 13 in comparison to healthy controls, as well as to participants who experience abuse before the age of 13. None of these analyses yielded significant findings.

© 2023 Korgaonkar MS et al. *JAMA Network Open.*

eTable 1. Summary of motion parameters for entire dataset

variable	max	mean	sd
MeanFD	0.189	0.079	0.035
MaxTranslation_MM	5.688	1.143	0.697
MaxRotation_RD	5.675	1.199	0.727
PercentOutliers	41.389	8.008	8.797

© 2023 Korgaonkar MS et al. *JAMA Network Open.*

eTable 2. Summary of network-wise distribution of edges, difference between groups

	No Abuse		Abuse <13yrs		Summary of Edges by Network				*T* test	Effect Size
networks	mean	SD	mean	SD	Number Edges	Positive Edges	Negative Edges	% Edges	P.value	Cohen's D
ECN-SMN	0.040	0.101	0.118	0.104	70	37	33	26.82	<.001	0.78
VAN-SMN	0.052	0.107	0.131	0.120	66	46	20	25.29	<.001	0.72
DMN-DAN	-0.092	0.133	-0.016	0.130	13	2	11	4.98	<.001	0.57
DAN-SMN	0.086	0.125	0.162	0.135	32	28	4	12.26	<.001	0.60
DMN-SMN	-0.068	0.110	-0.006	0.110	46	4	42	17.62	<.001	0.56
ECN-DAN	0.224	0.205	0.324	0.189	4	4		1.53	<.001	0.50
VAN-VAN	0.327	0.145	0.394	0.150	4	4		1.53	<.001	0.46
VAN-DAN	0.127	0.138	0.191	0.145	4	4		1.53	<.001	0.46
SMN-SMN	0.201	0.157	0.271	0.195	10	10		3.83	<.001	0.42
SC-VAN	0.190	0.212	0.250	0.189	2	2		0.77	0.003	0.29
ECN-VAN	0.456	0.250	0.532	0.294	1	1		0.38	0.008	0.29
DMN-VAN	0.101	0.179	0.146	0.171	2	2		0.77	0.009	0.26
DAN-DAN	0.189	0.161	0.233	0.175	2	2		0.77	0.012	0.27
DAN-VN	0.209	0.185	0.249	0.181	5	4	1	1.92	0.031	0.22

© 2023 Korgaonkar MS et al. *JAMA Network Open.*

eTable 3. List of all significant connections identified in NBS as increased in participants that experienced child abuse

Region1_Network1	X	Y	Z	Region2_Network2	X	Y	Z	TestStat
**DAN-DAN**								
69_DorsAttn_Post_1_L	-44	-42	-22	71_DorsAttn_Post_3_L	-56	-62	0	3.55
70_DorsAttn_Post_2_L	-48	-56	-16	291_DorsAttn_FEF_2_R	28	-4	52	3.61
**DAN-SMN**								
235_SomMot_5_R	38	-8	14	291_DorsAttn_FEF_2_R	28	-4	52	3.61
87_DorsAttn_FEF_2_L	-26	0	56	245_SomMot_15_R	60	6	30	3.4
87_DorsAttn_FEF_2_L	-26	0	56	246_SomMot_16_R	60	-6	26	3.3
89_DorsAttn_FEF_4_L	-22	6	64	245_SomMot_15_R	60	6	30	4.15
89_DorsAttn_FEF_4_L	-22	6	64	246_SomMot_16_R	60	-6	26	3.84
89_DorsAttn_FEF_4_L	-22	6	64	248_SomMot_18_R	52	-6	38	3.77
89_DorsAttn_FEF_4_L	-22	6	64	251_SomMot_21_R	52	-12	50	3.36
90_DorsAttn_PrCv_1_L	-50	6	26	251_SomMot_21_R	52	-12	50	4.19
90_DorsAttn_PrCv_1_L	-50	6	26	253_SomMot_23_R	50	-26	56	3.37
239_SomMot_9_R	42	-14	18	291_DorsAttn_FEF_2_R	28	-4	52	3.57
241_SomMot_11_R	50	-10	14	291_DorsAttn_FEF_2_R	28	-4	52	3.67
241_SomMot_11_R	50	-10	14	293_DorsAttn_PrCv_1_R	48	8	24	3.54
246_SomMot_16_R	60	-6	26	291_DorsAttn_FEF_2_R	28	-4	52	3.36
35_SomMot_4_L	-38	-8	12	291_DorsAttn_FEF_2_R	28	-4	52	3.5
35_SomMot_4_L	-38	-8	12	87_DorsAttn_FEF_2_L	-26	0	56	4.25
35_SomMot_4_L	-38	-8	12	88_DorsAttn_FEF_3_L	-30	-8	52	3.44
37_SomMot_6_L	-58	-36	16	279_DorsAttn_Post_9_R	46	-28	42	3.4
38_SomMot_7_L	-40	-36	14	291_DorsAttn_FEF_2_R	28	-4	52	3.7
38_SomMot_7_L	-40	-36	14	88_DorsAttn_FEF_3_L	-30	-8	52	3.37
39_SomMot_8_L	-48	-12	14	291_DorsAttn_FEF_2_R	28	-4	52	3.51
39_SomMot_8_L	-48	-12	14	87_DorsAttn_FEF_2_L	-26	0	56	3.42
40_SomMot_9_L	-60	-2	10	87_DorsAttn_FEF_2_L	-26	0	56	3.81
40_SomMot_9_L	-60	-2	10	88_DorsAttn_FEF_3_L	-30	-8	52	3.41
43_SomMot_12_L	-60	-2	24	87_DorsAttn_FEF_2_L	-26	0	56	3.72
43_SomMot_12_L	-60	-2	24	89_DorsAttn_FEF_4_L	-22	6	64	4.22
44_SomMot_13_L	-62	-10	32	89_DorsAttn_FEF_4_L	-22	6	64	4.38
45_SomMot_14_L	-54	-8	30	87_DorsAttn_FEF_2_L	-26	0	56	3.63
45_SomMot_14_L	-54	-8	30	89_DorsAttn_FEF_4_L	-22	6	64	4.3
47_SomMot_16_L	-52	-6	44	89_DorsAttn_FEF_4_L	-22	6	64	4.52
50_SomMot_19_L	-40	-14	48	293_DorsAttn_PrCv_1_R	48	8	24	3.36

© 2023 Korgaonkar MS et al. *JAMA Network Open.*

50_SomMot_19_L	-40	-14	48	88_DorsAttn_FEF_3_L	-30	-8	52	3.39
51_SomMot_20_L	-48	-18	54	90_DorsAttn_PrCv_1_L	-50	6	26	3.3
**VAN-DAN**								
107_SalVentAttn_Med_1_L	-6	22	32	279_DorsAttn_Post_9_R	46	-28	42	3.6
87_DorsAttn_FEF_2_L	-26	0	56	102_SalVentAttn_FrOper_6_L	-36	4	10	3.67
87_DorsAttn_FEF_2_L	-26	0	56	305_SalVentAttn_FrOper_4_R	40	-2	6	3.33
92_SalVentAttn_ParOper_1_L	-54	-32	22	278_DorsAttn_Post_8_R	44	-38	50	3.32
**DAN-ECN**								
146_Cont_Cing_1_L	-4	6	28	278_DorsAttn_Post_8_R	44	-38	50	3.61
146_Cont_Cing_1_L	-4	6	28	279_DorsAttn_Post_9_R	46	-28	42	4.3
278_DorsAttn_Post_8_R	44	-38	50	359_Cont_Cing_2_R	4	2	30	3.67
279_DorsAttn_Post_9_R	46	-28	42	359_Cont_Cing_2_R	4	2	30	4.29
**DAN-DMN**								
153_Default_Temp_5_L	-52	6	-12	291_DorsAttn_FEF_2_R	28	-4	52	3.53
153_Default_Temp_5_L	-52	6	-12	292_DorsAttn_FEF_3_R	24	-2	64	3.48
173_Default_PFC_8_L	-6	60	6	279_DorsAttn_Post_9_R	46	-28	42	3.33
174_Default_PFC_9_L	-6	44	6	278_DorsAttn_Post_8_R	44	-38	50	3.43
174_Default_PFC_9_L	-6	44	6	279_DorsAttn_Post_9_R	46	-28	42	3.62
177_Default_PFC_12_L	-6	34	20	278_DorsAttn_Post_8_R	44	-38	50	3.74
177_Default_PFC_12_L	-6	34	20	279_DorsAttn_Post_9_R	46	-28	42	3.94
276_DorsAttn_Post_6_R	58	-22	44	382_Default_PFCm_4_R	8	54	12	3.39
278_DorsAttn_Post_8_R	44	-38	50	382_Default_PFCm_4_R	8	54	12	3.78
278_DorsAttn_Post_8_R	44	-38	50	384_Default_PFCm_6_R	6	26	18	3.33
279_DorsAttn_Post_9_R	46	-28	42	382_Default_PFCm_4_R	8	54	12	4.23
279_DorsAttn_Post_9_R	46	-28	42	384_Default_PFCm_6_R	6	26	18	3.52
76_DorsAttn_Post_8_L	-46	-30	44	377_Default_PFCv_3_R	48	32	-8	3.38
**DAN-VIS**								
23_Vis_23_L	-40	-84	14	69_DorsAttn_Post_1_L	-44	-42	-22	3.85
69_DorsAttn_Post_1_L	-44	-42	-22	222_Vis_22_R	44	-78	10	3.34
27_Vis_27_L	-26	-84	22	69_DorsAttn_Post_1_L	-44	-42	-22	3.43
27_Vis_27_L	-26	-84	22	70_DorsAttn_Post_2_L	-48	-56	-16	3.47
8_Vis_8_L	-46	-70	-8	70_DorsAttn_Post_2_L	-48	-56	-16	3.5
**SMN-SMN**								
238_SomMot_8_R	64	-34	10	255_SomMot_25_R	44	-22	54	3.39
37_SomMot_6_L	-58	-36	16	249_SomMot_19_R	54	-16	40	3.45
37_SomMot_6_L	-58	-36	16	251_SomMot_21_R	52	-12	50	3.73
37_SomMot_6_L	-58	-36	16	253_SomMot_23_R	50	-26	56	4.24
37_SomMot_6_L	-58	-36	16	255_SomMot_25_R	44	-22	54	3.76

© 2023 Korgaonkar MS et al. *JAMA Network Open.*

37_SomMot_6_L	-58	-36	16	51_SomMot_20_L	-48	-18	54	3.37
37_SomMot_6_L	-58	-36	16	52_SomMot_21_L	-48	-30	58	4.34
37_SomMot_6_L	-58	-36	16	60_SomMot_29_L	-30	-38	66	3.67
37_SomMot_6_L	-58	-36	16	65_SomMot_34_L	-14	-12	72	3.8
38_SomMot_7_L	-40	-36	14	56_SomMot_25_L	-4	-8	60	3.36
**SMN-VAN**								
105_SalVentAttn_FrOper_9_L	-52	8	14	251_SomMot_21_R	52	-12	50	3.99
105_SalVentAttn_FrOper_9_L	-52	8	14	253_SomMot_23_R	50	-26	56	3.6
107_SalVentAttn_Med_1_L	-6	22	32	232_SomMot_2_R	62	-18	0	3.57
107_SalVentAttn_Med_1_L	-6	22	32	233_SomMot_3_R	54	-14	6	3.44
107_SalVentAttn_Med_1_L	-6	22	32	234_SomMot_4_R	40	-20	4	3.89
107_SalVentAttn_Med_1_L	-6	22	32	235_SomMot_5_R	38	-8	14	3.73
107_SalVentAttn_Med_1_L	-6	22	32	239_SomMot_9_R	42	-14	18	3.53
107_SalVentAttn_Med_1_L	-6	22	32	241_SomMot_11_R	50	-10	14	4.44
107_SalVentAttn_Med_1_L	-6	22	32	246_SomMot_16_R	60	-6	26	4.04
107_SalVentAttn_Med_1_L	-6	22	32	248_SomMot_18_R	52	-6	38	3.65
107_SalVentAttn_Med_1_L	-6	22	32	251_SomMot_21_R	52	-12	50	3.6
107_SalVentAttn_Med_1_L	-6	22	32	253_SomMot_23_R	50	-26	56	3.57
107_SalVentAttn_Med_1_L	-6	22	32	255_SomMot_25_R	44	-22	54	3.85
107_SalVentAttn_Med_1_L	-6	22	32	256_SomMot_26_R	38	-20	64	3.52
107_SalVentAttn_Med_1_L	-6	22	32	257_SomMot_27_R	32	-34	64	3.54
107_SalVentAttn_Med_1_L	-6	22	32	259_SomMot_29_R	34	-28	62	3.83
107_SalVentAttn_Med_1_L	-6	22	32	264_SomMot_34_R	22	-28	68	3.33
108_SalVentAttn_Med_2_L	-6	0	40	241_SomMot_11_R	50	-10	14	3.36
110_SalVentAttn_Med_4_L	-6	10	48	239_SomMot_9_R	42	-14	18	3.49
235_SomMot_5_R	38	-8	14	311_SalVentAttn_Med_1_R	8	18	36	3.9
237_SomMot_7_R	60	-24	10	300_SalVentAttn_TempOccPar_7_R	62	-38	36	3.61
239_SomMot_9_R	42	-14	18	311_SalVentAttn_Med_1_R	8	18	36	3.98
241_SomMot_11_R	50	-10	14	310_SalVentAttn_PFCl_1_R	32	44	28	3.47
241_SomMot_11_R	50	-10	14	311_SalVentAttn_Med_1_R	8	18	36	4.22
246_SomMot_16_R	60	-6	26	306_SalVentAttn_FrOper_5_R	36	22	4	3.58
246_SomMot_16_R	60	-6	26	311_SalVentAttn_Med_1_R	8	18	36	3.83
253_SomMot_23_R	50	-26	56	296_SalVentAttn_TempOccPar_3_R	62	-40	22	3.35
253_SomMot_23_R	50	-26	56	297_SalVentAttn_TempOccPar_4_R	58	-32	24	3.38
34_SomMot_3_L	-36	-24	10	107_SalVentAttn_Med_1_L	-6	22	32	3.59
35_SomMot_4_L	-38	-8	12	99_SalVentAttn_FrOper_3_L	-32	24	0	3.75
38_SomMot_7_L	-40	-36	14	107_SalVentAttn_Med_1_L	-6	22	32	3.66
38_SomMot_7_L	-40	-36	14	108_SalVentAttn_Med_2_L	-6	0	40	3.51

38_SomMot_7_L	-40	-36	14	300_SalVentAttn_TempOccPar_7_R	62	-38	36	3.74
39_SomMot_8_L	-48	-12	14	107_SalVentAttn_Med_1_L	-6	22	32	4.13
39_SomMot_8_L	-48	-12	14	108_SalVentAttn_Med_2_L	-6	0	40	3.46
39_SomMot_8_L	-48	-12	14	300_SalVentAttn_TempOccPar_7_R	62	-38	36	3.8
39_SomMot_8_L	-48	-12	14	310_SalVentAttn_PFCl_1_R	32	44	28	3.51
39_SomMot_8_L	-48	-12	14	311_SalVentAttn_Med_1_R	8	18	36	3.78
39_SomMot_8_L	-48	-12	14	99_SalVentAttn_FrOper_3_L	-32	24	0	4.3
41_SomMot_10_L	-48	-24	18	107_SalVentAttn_Med_1_L	-6	22	32	3.47
41_SomMot_10_L	-48	-24	18	300_SalVentAttn_TempOccPar_7_R	62	-38	36	3.6
41_SomMot_10_L	-48	-24	18	99_SalVentAttn_FrOper_3_L	-32	24	0	3.69
43_SomMot_12_L	-60	-2	24	107_SalVentAttn_Med_1_L	-6	22	32	3.8
43_SomMot_12_L	-60	-2	24	312_SalVentAttn_Med_2_R	8	2	42	3.4
46_SomMot_15_L	-54	-20	40	99_SalVentAttn_FrOper_3_L	-32	24	0	3.57
48_SomMot_17_L	-48	-16	40	105_SalVentAttn_FrOper_9_L	-52	8	14	3.51
48_SomMot_17_L	-48	-16	40	306_SalVentAttn_FrOper_5_R	36	22	4	3.36
49_SomMot_18_L	-8	-16	48	306_SalVentAttn_FrOper_5_R	36	22	4	3.3
51_SomMot_20_L	-48	-18	54	105_SalVentAttn_FrOper_9_L	-52	8	14	3.48
51_SomMot_20_L	-48	-18	54	92_SalVentAttn_ParOper_1_L	-54	-32	22	3.47
51_SomMot_20_L	-48	-18	54	99_SalVentAttn_FrOper_3_L	-32	24	0	3.78
52_SomMot_21_L	-48	-30	58	105_SalVentAttn_FrOper_9_L	-52	8	14	3.61
52_SomMot_21_L	-48	-30	58	107_SalVentAttn_Med_1_L	-6	22	32	3.74
52_SomMot_21_L	-48	-30	58	296_SalVentAttn_TempOccPar_3_R	62	-40	22	3.59
52_SomMot_21_L	-48	-30	58	297_SalVentAttn_TempOccPar_4_R	58	-32	24	3.61
52_SomMot_21_L	-48	-30	58	92_SalVentAttn_ParOper_1_L	-54	-32	22	3.95
52_SomMot_21_L	-48	-30	58	93_SalVentAttn_ParOper_2_L	-58	-44	28	3.67
52_SomMot_21_L	-48	-30	58	99_SalVentAttn_FrOper_3_L	-32	24	0	4.15
53_SomMot_22_L	-38	-24	52	297_SalVentAttn_TempOccPar_4_R	58	-32	24	3.31
57_SomMot_26_L	-36	-20	64	92_SalVentAttn_ParOper_1_L	-54	-32	22	3.32
60_SomMot_29_L	-30	-38	66	299_SalVentAttn_TempOccPar_6_R	62	-26	38	3.36
60_SomMot_29_L	-30	-38	66	92_SalVentAttn_ParOper_1_L	-54	-32	22	3.92
60_SomMot_29_L	-30	-38	66	95_SalVentAttn_ParOper_4_L	-62	-36	34	3.33
65_SomMot_34_L	-14	-12	72	108_SalVentAttn_Med_2_L	-6	0	40	3.34
99_SalVentAttn_FrOper_3_L	-32	24	0	241_SomMot_11_R	50	-10	14	3.71
99_SalVentAttn_FrOper_3_L	-32	24	0	246_SomMot_16_R	60	-6	26	3.44
**SMN-ECN**								
139_Cont_PFCl_6_L	-42	38	22	241_SomMot_11_R	50	-10	14	3.81
146_Cont_Cing_1_L	-4	6	28	235_SomMot_5_R	38	-8	14	3.33
146_Cont_Cing_1_L	-4	6	28	239_SomMot_9_R	42	-14	18	3.41
146_Cont_Cing_1_L	-4	6	28	241_SomMot_11_R	50	-10	14	3.8

© 2023 Korgaonkar MS et al. *JAMA Network Open.*

146_Cont_Cing_1_L	-4	6	28	246_SomMot_16_R	60	-6	26	3.98
146_Cont_Cing_1_L	-4	6	28	248_SomMot_18_R	52	-6	38	3.98
146_Cont_Cing_1_L	-4	6	28	249_SomMot_19_R	54	-16	40	3.5
146_Cont_Cing_1_L	-4	6	28	251_SomMot_21_R	52	-12	50	3.61
146_Cont_Cing_1_L	-4	6	28	253_SomMot_23_R	50	-26	56	4.25
146_Cont_Cing_1_L	-4	6	28	255_SomMot_25_R	44	-22	54	4.28
146_Cont_Cing_1_L	-4	6	28	256_SomMot_26_R	38	-20	64	3.69
146_Cont_Cing_1_L	-4	6	28	257_SomMot_27_R	32	-34	64	4.09
146_Cont_Cing_1_L	-4	6	28	259_SomMot_29_R	34	-28	62	4.14
146_Cont_Cing_1_L	-4	6	28	261_SomMot_31_R	28	-10	64	3.37
146_Cont_Cing_1_L	-4	6	28	264_SomMot_34_R	22	-28	68	3.41
235_SomMot_5_R	38	-8	14	351_Cont_PFCl_11_R	38	34	38	3.31
235_SomMot_5_R	38	-8	14	355_Cont_PFCl_15_R	24	10	58	3.73
235_SomMot_5_R	38	-8	14	359_Cont_Cing_2_R	4	2	30	3.3
235_SomMot_5_R	38	-8	14	361_Cont_PFCmp_2_R	4	28	48	3.44
239_SomMot_9_R	42	-14	18	355_Cont_PFCl_15_R	24	10	58	3.73
239_SomMot_9_R	42	-14	18	359_Cont_Cing_2_R	4	2	30	3.39
239_SomMot_9_R	42	-14	18	361_Cont_PFCmp_2_R	4	28	48	3.4
241_SomMot_11_R	50	-10	14	345_Cont_PFCl_5_R	42	46	14	3.6
241_SomMot_11_R	50	-10	14	347_Cont_PFCl_7_R	48	18	24	3.37
241_SomMot_11_R	50	-10	14	350_Cont_PFCl_10_R	38	10	34	3.66
241_SomMot_11_R	50	-10	14	351_Cont_PFCl_11_R	38	34	38	3.37
241_SomMot_11_R	50	-10	14	355_Cont_PFCl_15_R	24	10	58	4.28
241_SomMot_11_R	50	-10	14	359_Cont_Cing_2_R	4	2	30	4.02
241_SomMot_11_R	50	-10	14	360_Cont_PFCmp_1_R	8	34	24	3.39
241_SomMot_11_R	50	-10	14	361_Cont_PFCmp_2_R	4	28	48	3.69
246_SomMot_16_R	60	-6	26	340_Cont_PFCv_1_R	34	22	-8	3.39
246_SomMot_16_R	60	-6	26	355_Cont_PFCl_15_R	24	10	58	3.82
246_SomMot_16_R	60	-6	26	359_Cont_Cing_2_R	4	2	30	3.94
248_SomMot_18_R	52	-6	38	359_Cont_Cing_2_R	4	2	30	3.98
251_SomMot_21_R	52	-12	50	359_Cont_Cing_2_R	4	2	30	3.31
253_SomMot_23_R	50	-26	56	344_Cont_PFCl_4_R	50	40	6	3.33
253_SomMot_23_R	50	-26	56	359_Cont_Cing_2_R	4	2	30	4.21
255_SomMot_25_R	44	-22	54	358_Cont_Cing_1_R	6	-28	28	3.43
255_SomMot_25_R	44	-22	54	359_Cont_Cing_2_R	4	2	30	3.95
256_SomMot_26_R	38	-20	64	358_Cont_Cing_1_R	6	-28	28	3.66
256_SomMot_26_R	38	-20	64	359_Cont_Cing_2_R	4	2	30	3.78
257_SomMot_27_R	32	-34	64	359_Cont_Cing_2_R	4	2	30	4.19
259_SomMot_29_R	34	-28	62	359_Cont_Cing_2_R	4	2	30	4.16

259_SomMot_29_R	34	-28	62	360_Cont_PFCmp_1_R	8	34	24	3.34
261_SomMot_31_R	28	-10	64	359_Cont_Cing_2_R	4	2	30	3.88
263_SomMot_33_R	22	-24	66	359_Cont_Cing_2_R	4	2	30	3.43
264_SomMot_34_R	22	-28	68	359_Cont_Cing_2_R	4	2	30	3.75
269_SomMot_39_R	16	-18	74	359_Cont_Cing_2_R	4	2	30	3.38
35_SomMot_4_L	-38	-8	12	142_Cont_PFCl_9_L	-36	32	38	3.53
35_SomMot_4_L	-38	-8	12	351_Cont_PFCl_11_R	38	34	38	3.35
35_SomMot_4_L	-38	-8	12	355_Cont_PFCl_15_R	24	10	58	3.43
39_SomMot_8_L	-48	-12	14	146_Cont_Cing_1_L	-4	6	28	4.03
39_SomMot_8_L	-48	-12	14	351_Cont_PFCl_11_R	38	34	38	3.49
39_SomMot_8_L	-48	-12	14	355_Cont_PFCl_15_R	24	10	58	4.17
39_SomMot_8_L	-48	-12	14	359_Cont_Cing_2_R	4	2	30	4.09
43_SomMot_12_L	-60	-2	24	143_Cont_PFCv_1_L	-34	16	-8	3.69
43_SomMot_12_L	-60	-2	24	146_Cont_Cing_1_L	-4	6	28	3.64
43_SomMot_12_L	-60	-2	24	355_Cont_PFCl_15_R	24	10	58	3.36
43_SomMot_12_L	-60	-2	24	359_Cont_Cing_2_R	4	2	30	3.89
44_SomMot_13_L	-62	-10	32	143_Cont_PFCv_1_L	-34	16	-8	3.37
44_SomMot_13_L	-62	-10	32	355_Cont_PFCl_15_R	24	10	58	4.23
45_SomMot_14_L	-54	-8	30	146_Cont_Cing_1_L	-4	6	28	3.38
45_SomMot_14_L	-54	-8	30	359_Cont_Cing_2_R	4	2	30	3.72
48_SomMot_17_L	-48	-16	40	341_Cont_PFCl_1_R	28	54	-14	3.32
51_SomMot_20_L	-48	-18	54	346_Cont_PFCl_6_R	50	30	18	3.36
51_SomMot_20_L	-48	-18	54	351_Cont_PFCl_11_R	38	34	38	3.54
51_SomMot_20_L	-48	-18	54	355_Cont_PFCl_15_R	24	10	58	3.58
52_SomMot_21_L	-48	-30	58	340_Cont_PFCv_1_R	34	22	-8	3.53
65_SomMot_34_L	-14	-12	72	146_Cont_Cing_1_L	-4	6	28	3.34
65_SomMot_34_L	-14	-12	72	359_Cont_Cing_2_R	4	2	30	3.61
**SMN-DMN**								
167_Default_PFC_2_L	-36	36	-12	251_SomMot_21_R	52	-12	50	3.6
167_Default_PFC_2_L	-36	36	-12	253_SomMot_23_R	50	-26	56	4.14
167_Default_PFC_2_L	-36	36	-12	255_SomMot_25_R	44	-22	54	3.57
167_Default_PFC_2_L	-36	36	-12	256_SomMot_26_R	38	-20	64	3.58
167_Default_PFC_2_L	-36	36	-12	259_SomMot_29_R	34	-28	62	3.48
170_Default_PFC_5_L	-46	32	-10	241_SomMot_11_R	50	-10	14	3.45
170_Default_PFC_5_L	-46	32	-10	251_SomMot_21_R	52	-12	50	3.42
170_Default_PFC_5_L	-46	32	-10	253_SomMot_23_R	50	-26	56	3.38
172_Default_PFC_7_L	-48	28	0	251_SomMot_21_R	52	-12	50	3.65
172_Default_PFC_7_L	-48	28	0	253_SomMot_23_R	50	-26	56	3.95
172_Default_PFC_7_L	-48	28	0	255_SomMot_25_R	44	-22	54	3.37

172_Default_PFC_7_L	-48	28	0	256_SomMot_26_R	38	-20	64	3.35
172_Default_PFC_7_L	-48	28	0	257_SomMot_27_R	32	-34	64	3.4
177_Default_PFC_12_L	-6	34	20	253_SomMot_23_R	50	-26	56	3.5
177_Default_PFC_12_L	-6	34	20	255_SomMot_25_R	44	-22	54	3.51
246_SomMot_16_R	60	-6	26	384_Default_PFCm_6_R	6	26	18	3.69
248_SomMot_18_R	52	-6	38	384_Default_PFCm_6_R	6	26	18	3.37
251_SomMot_21_R	52	-12	50	378_Default_PFCv_4_R	54	24	6	3.43
253_SomMot_23_R	50	-26	56	377_Default_PFCv_3_R	48	32	-8	3.74
253_SomMot_23_R	50	-26	56	378_Default_PFCv_4_R	54	24	6	3.47
253_SomMot_23_R	50	-26	56	382_Default_PFCm_4_R	8	54	12	3.96
255_SomMot_25_R	44	-22	54	377_Default_PFCv_3_R	48	32	-8	3.49
255_SomMot_25_R	44	-22	54	382_Default_PFCm_4_R	8	54	12	3.76
255_SomMot_25_R	44	-22	54	384_Default_PFCm_6_R	6	26	18	3.5
256_SomMot_26_R	38	-20	64	382_Default_PFCm_4_R	8	54	12	3.45
259_SomMot_29_R	34	-28	62	377_Default_PFCv_3_R	48	32	-8	3.69
35_SomMot_4_L	-38	-8	12	167_Default_PFC_2_L	-36	36	-12	3.35
35_SomMot_4_L	-38	-8	12	170_Default_PFC_5_L	-46	32	-10	3.34
46_SomMot_15_L	-54	-20	40	377_Default_PFCv_3_R	48	32	-8	3.46
46_SomMot_15_L	-54	-20	40	378_Default_PFCv_4_R	54	24	6	3.47
48_SomMot_17_L	-48	-16	40	376_Default_PFCv_2_R	36	38	-14	3.34
48_SomMot_17_L	-48	-16	40	377_Default_PFCv_3_R	48	32	-8	3.62
48_SomMot_17_L	-48	-16	40	378_Default_PFCv_4_R	54	24	6	3.79
50_SomMot_19_L	-40	-14	48	375_Default_PFCv_1_R	34	22	-18	3.41
50_SomMot_19_L	-40	-14	48	378_Default_PFCv_4_R	54	24	6	3.38
51_SomMot_20_L	-48	-18	54	377_Default_PFCv_3_R	48	32	-8	3.49
51_SomMot_20_L	-48	-18	54	378_Default_PFCv_4_R	54	24	6	4.37
52_SomMot_21_L	-48	-30	58	172_Default_PFC_7_L	-48	28	0	3.51
52_SomMot_21_L	-48	-30	58	377_Default_PFCv_3_R	48	32	-8	3.75
52_SomMot_21_L	-48	-30	58	378_Default_PFCv_4_R	54	24	6	4.43
53_SomMot_22_L	-38	-24	52	377_Default_PFCv_3_R	48	32	-8	3.44
53_SomMot_22_L	-38	-24	52	378_Default_PFCv_4_R	54	24	6	3.32
57_SomMot_26_L	-36	-20	64	378_Default_PFCv_4_R	54	24	6	3.39
59_SomMot_28_L	-32	-30	62	172_Default_PFC_7_L	-48	28	0	3.43
59_SomMot_28_L	-32	-30	62	378_Default_PFCv_4_R	54	24	6	3.39
60_SomMot_29_L	-30	-38	66	172_Default_PFC_7_L	-48	28	0	3.37
**VAN-VAN**								
107_SalVentAttn_Med_1_L	-6	22	32	305_SalVentAttn_FrOper_4_R	40	-2	6	3.68
108_SalVentAttn_Med_2_L	-6	0	40	295_SalVentAttn_TempOccPar_2_R	52	-40	12	3.49
98_SalVentAttn_FrOper_2_L	-38	2	-4	99_SalVentAttn_FrOper_3_L	-32	24	0	3.4

© 2023 Korgaonkar MS et al. *JAMA Network Open.*

99_SalVentAttn_FrOper_3_L	-32	24	0	102_SalVentAttn_FrOper_6_L	-36	4	10	3.35
**VAN-ECN**								
297_SalVentAttn_TempOccPar_4_R	58	-32	24	335_Cont_Par_4_R	54	-32	52	3.43
**VAN-DMN**								
108_SalVentAttn_Med_2_L	-6	0	40	152_Default_Temp_4_L	-56	-8	-14	3.33
110_SalVentAttn_Med_4_L	-6	10	48	371_Default_Temp_5_R	64	-24	-8	3.32
**VAN-Subcortical**								
107_SalVentAttn_Med_1_L	-6	22	32	420_BGPu_R	29	-3	1	4.05
311_SalVentAttn_Med_1_R	8	18	36	420_BGPu_R	29	-3	1	3.78

eTable 4. Proportion of abuse types at different ages of onset

	Abused 0 to 3	***Abused 4 to 7***	***Abused 8 to 12***	***Abused 13 to 17***
Total	12	42	73	78
Percentage	5.8	20.5	35.6	38.0
Sex Abuse				
Total	5	19	25	18
Percentage	41.6	45.2	34.2	23.0
Physical Abuse				
Total	6	22	40	33
Percentage	50.0	52.4	54.8	42.3
Emotional Abuse				
Total	7	23	61	67
Percentage	58.3	54.7	83.5	85.9

eReferences

1. McFarlane A, Clark CR, Bryant RA, Williams LM, Niaura R, Paul RH, Hitsman BL, Stroud L, Alexander DM, Gordon E. The impact of early life stress on psychophysiological, personality and behavioral measures in 740 non-clinical subjects. J*ournal of Integrative Neuroscience*, 2005; 4(1):27-40

2. Chu DA, Williams LM, Harris AW, Bryant RA, Gatt JM. Early life trauma predicts self-reported levels of depressive and anxiety symptoms in nonclinical community adults: relative contributions of early life stressor types and adult trauma exposure. *J Psychiatr Res*. 2013; 47(1):23-32.

3. Ball TM, Goldstein-Piekarski AN, Gatt JM, Williams LM. Quantifying person-level brain network functioning to facilitate clinical translation. *Transl Psychiatry*. 2017;7(10):e1248. doi:10.1038/tp.2017.204

4. Korgaonkar MS, Fornito A, Williams LM, Grieve SM. Abnormal structural networks characterize major depressive disorder: a connectome analysis. *Biol Psychiatry*. 2014;76(7):567-574. doi:10.1016/j.biopsych.2014.02.018

5. Achaibou A, Loth E, Bishop SJ. Distinct frontal and amygdala correlates of change detection for facial identity and expression. *Soc Cogn Affect Neurosci*. 2016;11. doi:10.1093/scan/nsv104

6. Goldstein-Piekarski AN, Staveland BR, Ball TM, Yesavage J, Korgaonkar MS, Williams LM. Intrinsic functional connectivity predicts remission on antidepressants: a randomized controlled trial to identify clinically applicable imaging biomarkers. *Transl Psychiatry*. 2018;8(1):1-11. doi:10.1038/s41398-018-0100-3

7. Power JD. Methods to detect, characterize, and remove motion artifact in resting state fMRI. *Neuroimage*. 2014;84. doi:10.1016/j.neuroimage.2013.08.048

8. Power JD, Barnes KA, Snyder AZ, Schlaggar BL, Petersen SE. Spurious but systematic correlations in functional connectivity MRI networks arise from subject motion. *Neuroimage*. 2012;59. doi:10.1016/j.neuroimage.2011.10.018

9. Friston KJ, Williams S, Howard R, Frackowiak RS, Turner R. Movement-related effects in fMRI time-series. *Magn Reson Med*. 1996;35. doi:10.1002/mrm.1910350312

© 2023 Korgaonkar MS et al. *JAMA Network Open.*
