# Supplementary material for: Association of Neural Connectome With Early Experiences of Abuse in Adults
Source: JAMA Netw Open. 2023 Jan 26;6(1):e2253082. doi: 10.1001/jamanetworkopen.2022.53082 (PMC9880798; doi:10.1001/jamanetworkopen.2022.53082)
Supplement: Supplement 2. — Data Sharing Statement [file jamanetwopen-e2253082-s002.pdf]

Data Sharing Statement

Korgaonkar. Association of Neural Connectome With Early Experiences of Abuse in Adults. *JAMA Netw Open*. Published January 26, 2023. doi:10.1001/jamanetworkopen.2022.53082

Data
Data available: Yes
Data types: Deidentified participant data, Data dictionary
How to access data: Data can be requested by email to Dr Mayuresh Korgaonkar (M.Korgaonkar@sydney.edu.au) or Prof Richard Bryant (r.bryant@nsw.edu.au)
When available: With publication

Supporting Documents
Document types: None

Additional Information
Who can access the data: researchers whose proposed use of the data has been approved
Types of analyses: for a specified purpose
Mechanisms of data availability: with a signed data access agreement
Any additional restrictions: Some of the data is industry sponsored and requires permission from sponsor prior to access.
